# The Sins of the Binaural Stethescope

**Published:** 1902-02-22

**Authors:** 


### THE SINS OF THE BINAURAL STETHESCOPE.

Dr. W. H. Syers[1] runs a tilt at the binaural stethescope, to the use of which, especially by young students who have not yet learned what to hear and what to listen for, he attributes the decay of the art of auscultation which he holds to be characteristic of present-day practitioners. He has convinced himself that as regards the investigation of the sounds of the heart, the binaural is most unreliable, and increasing experience not only of students' work, but of that of qualified practitioners who are in the habit of employing the double stethescope, confirms him in the opinion that unless the use of this instrument be discontinued, in no long time aortic regurgitation will be a disease which cannot be certainly diagnosed. In saying this, of course he does not refer to obvious cases, but to that very large class of murmurs due to incompetent aortic valves in which the bruit is soft, low-pitched, and not by any means easy to hear. It is these murmurs which are constantly passed over, the second sound being described as "clear." This is a most dangerous error, for it is well known that these are just the murmurs which are found in cases subsequently dying suddenly, the very softness of the murmur being a most unfavourable indication, as depending upon a feeble ventricular systole. To his certain knowledge, he says, this sort of murmur is being overlooked every day, and this not from want of accuracy of ear, but from the inherent defects of the untrustworthy instrument which is nowadays almost exclusively used. As regards the diagnosis of diseases of the heart, then, there is no middle course, "the double stethescope should be altogether done away with and abolished off the face of the earth." Again, in regard to the examination of the lungs there is nothing good to be said of this instrument in its strictly auscultatory aspect, and he especially alludes to the difficulty of differentiating friction sounds and crepitation, which, he says, is absolutely impossible with this deceitful instrument. He even goes so far as to maintain that by its use the hearing power of the observer is injured, and all that he finds in its favour is that it is convenient in illnesses which render it difficult to move the patient in a manner suitable for examination by the wooden instrument, and that in the case of babies and young children it may often be advantageously employed. But he especially protests against young students being allowed to learn their work with the binaural stethescope, which he holds is the cause of much of the indifferent diagnosis of chest diseases which is so often observed at the present time,—as to all of which, we need hardly remark that there are plenty of people to be found who will entirely disagree with Dr. Syers.

[1] Lancet, Feb. 8.

